# Evaluation of Nystatin Containing Chitosan Hydrogels as Potential Dual Action Bio-Active Restorative Materials: *in Vitro* Approach

**DOI:** 10.3390/jfb5040259

**Published:** 2014-11-28

**Authors:** V. Tamara Perchyonok, Vanessa Reher, Shengmiao Zhang, Nicki Basson, Sias Grobler

**Affiliations:** 1 Research and Development Department, VTPCHEM PTY LTD., Southport 4215, Australia; 2 School of Dentistry and Oral Health, Gold Coast campus, Griffith University, QLD 4222, Australia; 3 Oral and Dental Research Institute, Faculty of Dentistry, University of the Western Cape, Private Bag X1, Tygerberg 7505, Cape Town, South Africa; 4 School of Material Science and Engineering, East China University of Science and Technology, 130 Meilong Road, Shanghai 200237, China

**Keywords:** chitosan, hydrogel, nystatin, reactive oxygen species, antioxidants, functional biomaterials, microbiological activity, percentage release

## Abstract

Healing is a specific biological process related to the general phenomenon of growth and tissue regeneration and is a process generally affected by several systemic conditions or as detrimental side-effects of chemotherapy- and radiotherapy-induced inflammation of the oral mucosa. The objectives of this study is to evaluate the novel chitosan based functional drug delivery systems, which can be successfully incorporated into “dual action bioactive restorative materials”, capable of inducing *in vitro* improved wound healing prototype and containing an antibiotic, such as nystatin, krill oil as an antioxidant and hydroxyapatite as a molecular bone scaffold, which is naturally present in bone and is reported to be successfully used in promoting bone integration when implanted as well as promoting healing. The hydrogels were prepared using a protocol as previously reported by us. The physico-chemical features, including surface morphology (SEM), release behaviors, stability of the therapeutic agent-antioxidant-chitosan, were measured and compared to the earlier reported chitosan-antioxidant containing hydrogels. Structural investigations of the reactive surface of the hydrogel are reported. Release of nystatin was investigated for all newly prepared hydrogels. Bio-adhesive studies were performed in order to assess the suitability of these designer materials. Free radical defense capacity of the biomaterials was evaluated using established *in vitro* model. The bio-adhesive capacity of the materials in the *in vitro* system was tested and quantified. It was found that the favorable synergistic effect of free radical built-in defense mechanism of the new functional materials increased sustainable bio-adhesion and therefore acted as a functional multi-dimensional restorative material with potential application in wound healing *in vitro*.

## 1. Introduction

Reactive oxygen species (ROS) are associated with all the stages of the healing process [1,2,3,4,5,6]. ROS are produced by the inflammatory cells and play an integral role during this process [7,8,9,10,11,12]. Antioxidants administration is beneficial for healing [13].

Bioadhesive polymers appear to be particularly attractive for the development of alternative etch free dentin bonding system with an added advantage of additional therapeutic delivery systems to improve intra-dental administration of therapeutic and prophylactic agents if necessary [10,11,12,13,14,15]. Chitosan, which is a biologically safe biopolymer, has been proposed as a bioadhesive polymer and are of continuous interest to us due to its unique properties and flexibility in a broad range of oral applications reported by others and us recently [16,17,18,19,20].

The objectives of this study is to evaluate the novel chitosan based functional drug delivery systems, which can be successfully incorporated into “dual action bioactive restorative materials” capable to induce *in vitro* improved wound healing prototype and containing common antibiotics, such as nystatin, krill oil as an antioxidant, hydroxyapatite as a molecular bone scaffold, which is naturally present in bone and is reported to be successfully used in promoting bone integration when implanted as well as promoting healing. 

## 2. Results and Discussion

**Figure 1:**
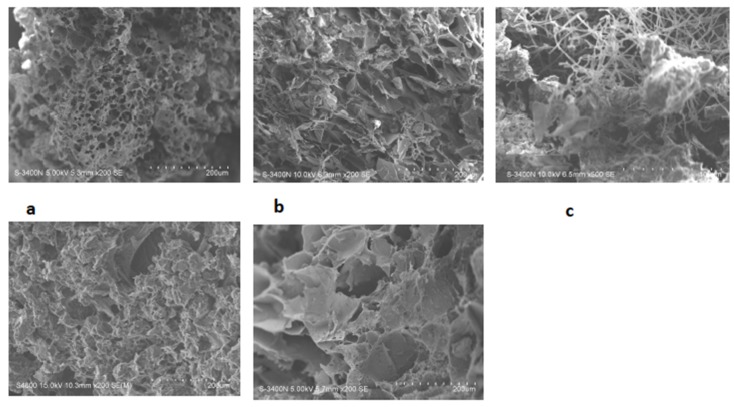
SEM photomicrograph of freeze-dried gels 1–5 (**a**–**e**).

The SEM images were obtained to characterize the microstructure of the freeze-dried gels and are presented in Figure 1. It could be seen that the gels displayed a homogeneous pore structure similar to a sponge. SEM analysis revealed interconnected pores of different sizes and flat, relatively smooth walls. The biomaterial remained intact after 24 days of immersion in artificial saliva as was confirmed by SEM. It was thought that the micro-porous structure of the gels could lead to high internal surface areas with low diffusional resistance in the gels. The pH of the prepared gels ranged from 5.46 to 6.94 (Table 1).

**Table 1:** Gel formulation prepared in the study.

Gel formulation *		Chitosan/Vitamin C (5:1) (w/w%)	Nystatin (w/w%)	Krill oil (w/w%)	Hydroxyapatite (w/w%)	pH
Ch/Vit C	Gel 1	5	0	0	0	5.46
Ch/Vit C/Nyst	Gel 2	5	1	0	0	6.84
Ch/Vit C/HA/Nyst	Gel 3	5	1	0	1	6.74
Ch/Vit C/Nyst/Kr	Gel 4	5	1	1	0	6.94
Ch/Vit C/Nyst/Kr/HA	Gel 5	5	1	1	1	6.65

* Ch, Chitosan; Vit C, vitamin C; Nyst, nystatin; HA, hydroxyapatite; Kr, krill oil.

**Figure 2:**
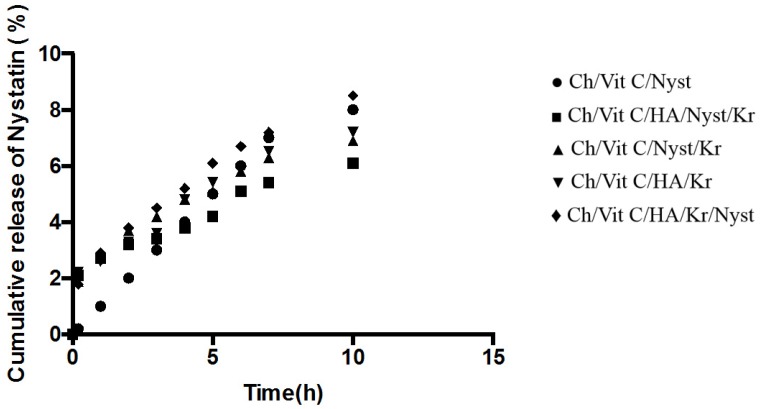
Cumulative percentage release of nystatin directly after manufacturing of the different hydrogels (*n* = 3, *p* < 0.05). Ch, Chitosan; Vit C, vitamin C; Nyst, nystatin; HA, hydroxyapatite; Kr, krill oil.

**Figure 3:**
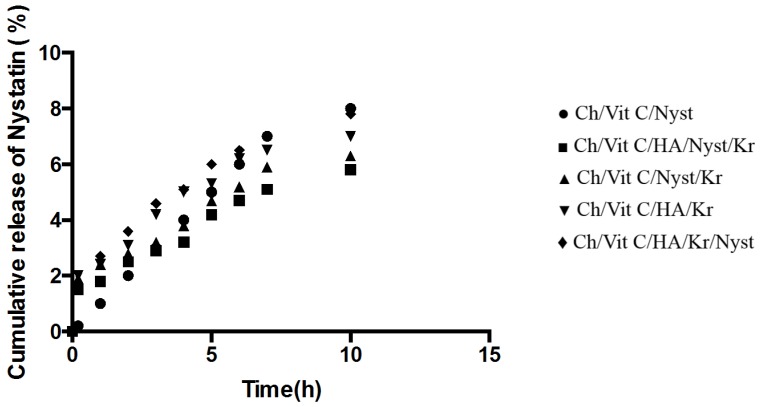
Percentage release of nystatin from the different hydrogels after 3 months storage at 21 °C (*n* = 3, *p* < 0.05).

The cumulative *in vitro* release of nystatin from the hydrogels, directly after manufacture and after three-month storage, is shown in Figure 2 and Figure 3, respectively. 

The *in vitro* release of therapeutic agents from the newly prepared hydrogel was carried out using USP dissolution apparatus type I. As the regression analysis of the obtained results for two kinetic models including zero order and Higushi’s model showed that Higushi’s model gave the highest value of *r*^2^ with significant difference (*p* < 0.05). Higushi’s model, where the cumulative amount of the released drug per unit area is proportional to the square root of time, is the more suitable model to describe the release kinetics from the gel preparations examined in the present study. The release of therapeutic agents from the hydrogels was studied as demonstrated in Figure 2 and Figure 3. The release of the corresponding hydrogels containing nystatin as a potential therapeutic agent prototype remained stable in the early hours of the experiment, allowing a more constant release, which would ensure an effective and prolonged anti-microbial activity when applied clinically. This property would make the system a suitable candidate for further development as a functional dual action restorative material.

### 2.1. Gel Stability

The results suggest that there is no significant decomposition observed after six-month storage at room temperature (21 °C), as antioxidant capacity of the materials stored for six months showed no diminished capacity compared to the freshly prepared hydrogels.

### 2.2. Studies of Equilibrium Swelling in the Hydrogels

**Figure 4:**
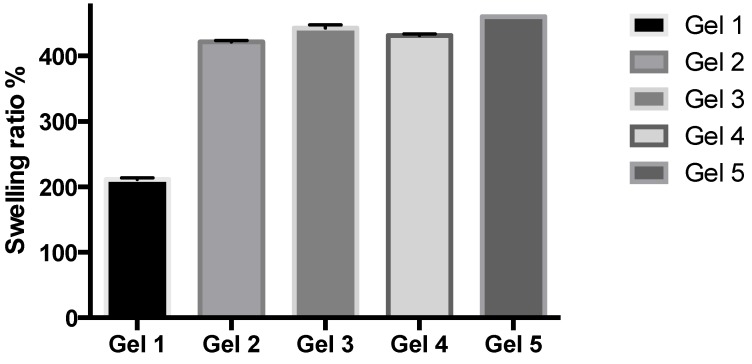
Swelling ratio (%) of the investigated hydrogels.

The hydrogels remained in the cylindrical form after swelling (Figure 4). Compared with dry state hydrogels, the swollen state hydrogel volume displayed significant increases.

### 2.3. Free Radical Defense Capability of the Prepared Hydrogels.

When a wound occurs, it is generally accompanied by classical symptoms of inflammation, such as pain, redness and edema. In the inflammation stage, the main aim is the removal of debris, damage tissue, and bacteria by neutrophils and macrophages. These cells have a role in antimicrobial defense and debridement of devitalized tissue by production of proteolytic enzymes and reactive oxygen species [21]. The amount of uncontrolled ROS is the main cause of the inability of the healing process to continue and therefore it would be ideal to utilize the antioxidant capacity of the “designer” hydrogels to detect and fight the free radical excess. This has been assessed using a previously established model known as the biologic Fenton reaction through which the HO**^•^** free radical can be generated in the presence of H_2_O_2_ [22].

As reported earlier, protein cross-linking can be used as a model for detection of free radical activity and activation of “molecular defense forces” [23]. Bovine serum albumin (BSA), a completely water-soluble protein, can be polymerized by hydroxyl radicals generated by the Fenton reaction system of Fe^2+^/EDTA/H_2_O_2_/ascorbate. As a result, the protein loses its water-solubility and the polymerized product precipitates. The decrease in the concentration of the water-soluble protein can subsequently be detected [23].

**Figure 5:**
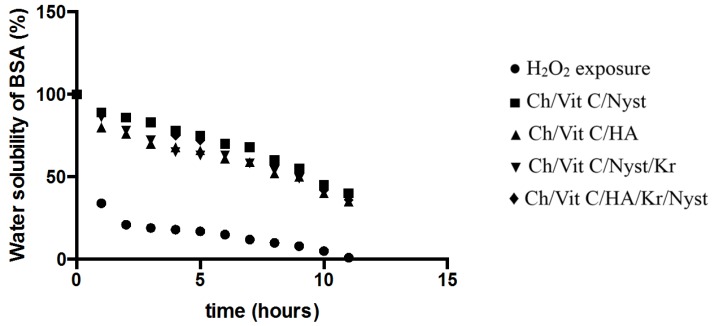
Insolubility of BSA exposed to the Fenton reaction system of Fe^2+^/EDTA/H_2_O_2_/ascorbate in the presence of the designer hydrogels *in vitro* free radical detection/defense prototype system in action (*n* = 3, *p* = 0.01).

We considered it worthwhile to study our “dual action bioactive restorative materials” as a “built-in defense mechanism” for the “site specific” counter reaction of the generated free radical production *in vitro*. Therefore we adopted the above-mentioned method for recording changes in water solubility of the BSA exposed to free radicals generated by an inorganic chemical system. As clearly demonstrated by Figure 5, upon exposure to standard H_2_O_2_ in the form of a Fe^2+^/EDTA/H_2_O_2_/ascorbate solution, which served as a base line for the determination of free radical generation under “prototype *in vitro free* radical damage”, upon incorporation of the chitosan substituted hydrogels, the built-in antioxidant capacity and therefore free radical defense of the *in vitro* model has been activated. This model represents the practical approach of *in situ* monitoring and testing of the amount of free radical production and synergistic antioxidant defense of the system. Further investigations and fine-tuning of the system are currently underway in our laboratory.

### 2.4. In Vitro Antifungal Activity of Novel Nystatin/Antioxidant Containing Chitosan Hydrogels

Although fungi are not primarily involved in the development of oral mucositis, they account for the most frequent infections of the damaged oral mucosa in immune-suppressed patients. Candidiasis is the predominant fungal infection manifesting itself by characteristic white coats or erythematous lesions in the corners of the mouth and on the soft palate and tongue [2]. In the present study, nystatin was selected because of its wide application, both locally and systemically, in treatment of oral fungal infections. 

Chitosan hydrogen scaffolds were designed in this study as carriers for antibiotics and showed a steady release of the medication. Three-dimensional chitosan matrices have been shown to be excellent tissue engineering scaffolds for cell attachment and growth. Chitosan has a scalloped structure and has been used in tissue engineering to culture hepatocytes, fibroblasts and cartilage cells because of its ability to promote cell attachment and growth [24,25,26,27,28]. In our investigation, chitosan was selected as the carrier for nystatin, mainly because it can both carry and deliver the medication, but also because it has other useful bioactivities such as antioxidant and anti-inflammatory properties [29]. 

**Figure 6:**
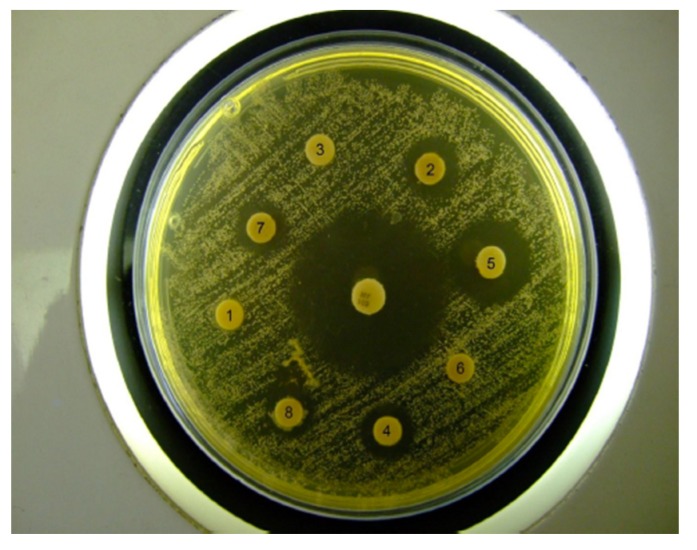
Example of *C. albicans* growth inhibition zones produced by the hydrogels tested.

Discs with additives but without nystatin gave no inhibition zones. The different preparations containing nystatin all gave clear inhibition zones with significant difference amongst them (Table 2). Furthermore, the test samples with nystatin and with additives all gave a significantly smaller inhibition zone than the nystatin antibiotic control disc although they contained nystatin at a higher concentration (Figure 6). This indicates that release of the nystatin from the formulations was inhibited to some extent, which is in accordance with previous results obtained from the nystatin release studies that showed that the cross-linked chitosan sponges were able to deliver active antibiotic for up to 10 days [16]. This slow release of the nystatin from the gel will be a beneficial effect that may enable a sustainable release over time.

**Table 2:** Diameters of Nystatin inhibition zones.

Sample	Diameters (mm)	
	Average (*n* = 3)	STD
Nystatin	20.17	0.3
Ch/Vit C/Nyst	10.08	0.23
Ch/Vit C/HA/Nyst	9.31	0.34
Ch/Vit C/HA/Nyst/Kr	8.36	0.33
Ch/Vit C/HA/Nyst/Kr/Fe^2+^	7.84	0.52
Ch/Vit C/HA/Nyst/Fe^2+^	8.91	0.33

### 2.5. Bio-Adhesion in Vitro Model

The term bio-adhesion refers to any bond formed between two biological surfaces or between a biological and a synthetic surface. In case of bio-adhesive drug delivery, the term bio-adhesion is used to describe the adhesion between polymers, either synthetic or natural, and soft tissues or the gastrointestinal mucosa [30]. In cases where the bond is formed with the mucus, the term muco-adhesion may be used synonymously with bio-adhesion [30]. Muco-adhesion can therefore be defined as a state in which two components, of which one is of biological origin, are held together for extended periods of time by the help of interfacial forces. Generally speaking, bio-adhesion is a term, which broadly includes adhesive interactions with any biological or biologically derived substance, and the term muco-adhesion is used when the bond is formed with a mucosal surface [30]. 

Over the last two decades, chitosan has been used for various biomedical and drug delivery applications due to its low toxicity, good biocompatibility and anti-microbial and bio-adhesive properties [31].

Higher adhesiveness of the gels is desired to maintain an intimate contact with the oral mucosa and preliminary *in vitro* results on the model adhesive surface (band-aid used as a prototype system) are summarized in Table 3. Chitosan hydrogels showed high adhesive force and this work of adhesion can be expected because of the well-known intrinsic bio-adhesive properties of chitosan. The adequate water absorption capacity together with the cationic nature, which promotes binding to the negative surface of the mucosa, can also explain these results [31]. 

According to Caffaggi, hydration of the polymer causes mobilization of the polymer chains and hence influences polymeric adhesion [32]. Appropriate swelling is important to guarantee adhesion, however, over hydration can form slippery non-adhesive hydrogels [33]. In addition the molecular arrangement of the polymeric chains, which are present in the new hydrogels with additives, such as nystatin, Krill oil, Vitamin C and hydroxyapatite, can further enable the hydrogel to interact with the substrate [31]. The correlation between the force and work of adhesion is noticeable for all. Further experiments are to be conducted to evaluate the bio-adhesive capacity of the designer hydrogels.

**Table 3:** Bio-adhesion testing *in vitro.* The presented values are an average (*n* = 5).

Hydrogel	Adhesive force (N) ± SD	Work of adhesion (N·cm) ± SD
Gel 1	1.20 ± 0.30	3.35 ± 0.48
Gel 2	1.12 ± 0.27	3.19 ± 0.52
Gel 3	1.01 ± 0.30	2.85 ± 0.41
Gel 4	1.15 ± 0.40	3.31 ± 0.31

### 2.6. Shear Bond Strength Tests for Dentin Bonding

**Figure 7:**
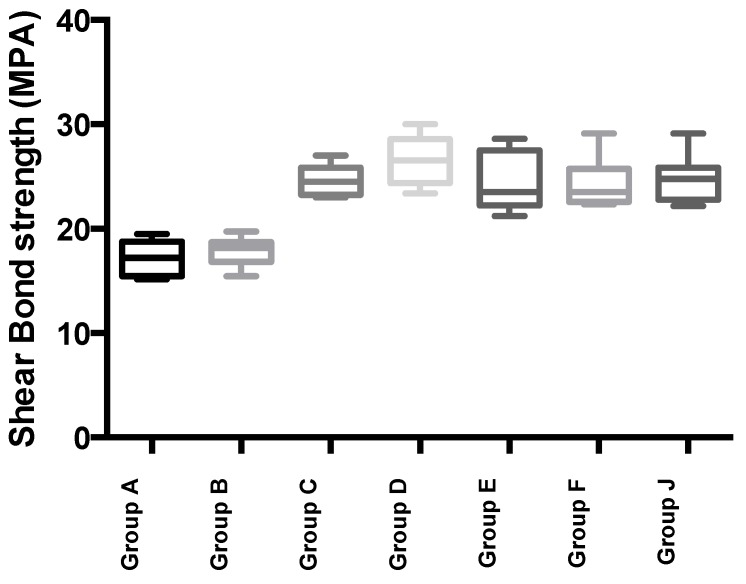
Shear bond strength of hydrogels after 24 hours of bonding to dentin, where Group A and Group B represent negative and positive control in the experiments, as described in Table 4

Mean shear bond strength values and difference between the groups are summarized in Figure 7 for bonding to dentin after 24 hours. In general there was an increase in bond strength of the dentin treated with the antioxidant (chitosan:Vit C complex) containing hydrogels compared to the bond strength of the conventionally bonded teeth. Interestingly, the increase in bond strength was also observed in the groups of hydrogen peroxide exposed samples and with no primer (conventional self etching bonding system commonly employed in restorative dentistry) used in the bonding exercises, suggesting that there additional benefits associated with chitosan:antioxidant system are in need of further investigations [31].

The results of this study suggests that the optimum results for the strengthening of dentin can be achieved throughout the immediate treatment with chitosan:nystatin “host:guest” complex with the increase of dentin bond strength. The additional advantage of the system may suggest that, antioxidant release from chitosan gel depends on the physical host:guest structure as well as pH properties and flexibilities of the material [32,33,34]. The additional benefit of using chitosan:nystatin:antioxidant system as a bonding/pre-bonding to enamel and dentin system lies in its ability to show favorable immediate results in terms of bonding effectiveness [27,34,35,36,37,38,39,40]. Therefore, the newly developed chitosan:antioxidant (combination of vitamin C and krill oil) systems, supporting our earlier reported results [27], are able to address the shortfalls affecting the long-term bonding performance of modern adhesives and addresses the current perspectives for improving bond durability of conventional adhesive systems.

## 3. Experimental Section

Chitosan:vitamin C (5:1) (Aldrich, Sydney, Australia), β-cyclodextrin (Aldrich, Sydney, Australia), glycerol (Sigma, Sydney, Australia), glacial acetic acid (E. Merck, Sydney, Australia) were used as received. The degree of de-acetylation of typical commercial chitosan used in this study is 87%. Chitosan with molecular weight 2.5 × 10^3^ KD was used in the study. The isoelectric point is 4.0–5.0. Resveratrol, β-carotene and propolis (Aurora Pharmaceuticals, Melbourne, Australia) were used as bought. 

### 3.1. Methods

#### 3.1.1. Preparation of the Various Antibiotic/Antioxidant Containing Hydrogels

Chitosan hydrogels were prepared using the methodology previously described [11,16]. Briefly, the antibiotic and antioxidant powders were incorporated into the hydrogels by dispersion of 0.2 g of the corresponding powder in glycerol using a mortar and pestle. Ten milliliters of a 5% (w/w) chitosan solution in glacial acetic acid was then added with continuous mixing to form the hydrogel. The strength of nystatin in the prepared gels was 100000 I.U. in each gram of the base. A summary of the newly prepared materials are highlighted in Table 1. 

#### 3.1.2. Determination of Gel pH

One gram of the prepared gel was accurately weighed and dispersed in 10 mL of distilled water. The pH of the dispersions was measured using a standard pH meter (HANNA instruments, HI8417, HANNA, Keysborough, Australia).

#### 3.1.3. *In Vitro* Nystatin Release

The release of nystatin from the gels was carried out with a United States Pharmacopeia (USP) dissolution apparatus type 1 (Copley Scientific, London, UK). In order to overcome the small volume of the dissolution medium, 100 mL beakers were used instead of the supplied jars. The basket of the dissolution apparatus was filled with 1 g of nystatin gel on a filter paper. The basket was immersed to about 1 cm of its surface in 50 mL of phosphate buffer at pH 6.8 and stirred at 100 rpm at 37 ± 0.5 °C. Twenty four samples of 2 mL each were collected at 0.2, 1, 2, 3, 4, 5, 6, 7, 8, 10, 15 and 24 h and the nystatin concentration in the samples was determined with a Ultraviolet (UV) spectrophotometer (Cintra 5, GBC Scientific Equipment, Melbourne, Australia) at a wavelength of 306 nm. Three replicate measurements were performed for each formulation. During the sampling process each sample volume was replaced by the same volume of phosphate buffer at pH 6.8 to maintain a constant volume and sink condition. 

#### 3.1.4. Microbiological Investigations

*Candida albicans* strain NCPF 3153 (National Collection of Pathogenic Fungi, UK) was used as test organism. The antifungal effectiveness of the prepared gels was measured using the standard Kirby-Bauer agar diffusion method [17]. Muller-Hinton agar (Oxoid, London, UK) plates were inoculated by streaking a standardized inoculum containing 10^7^–10^8^ CFU with a cotton swab. 5 mg of each hydrogel was applied to 6 mm diameter paper discs (500 I.U./disc). The paper discs were placed on the Muller-Hinton agar medium and incubated at 37 °C for 24 h. The effectiveness of the prepared gels was compared to chitosan gel containing no nystatin and an antibiotic sensitivity disc (Mast Laboratories, Merseyside, UK) containing 100 I.U. of nystatin/disc. The diameter of the zones of growth inhibition was measured from 3 different angles with a caliper. Each combination of additive was tested in triplicate. 

#### 3.1.5. Bio-Adhesive Investigation

Bio-adhesion studies were done using a Chatillon apparatus (Chatillon, Ametek, Largo, FL, USA) for force measurement. This method determines the maximum force and work needed to separate two surfaces in intimate contact. The hydrogels (0.1 g) were homogeneously spread on a 1 cm^2^ glass disc and then the discs were fixed to the support of the tensile strength tester using double side adhesive. The gel was brought into contact with a commercially available Band-Aid strip in order to simulate skin attachment. After a preset contact time of 1 min under contact strength of 0.5 N, the 2 surfaces were separated at a constant rate of displacement at 1 mm/s. The strength was recorded as a function of the displacement, which allowed determination of the maximal detachment force *F*_max_ and the work of adhesion *W*, which was calculated from the area under the strength-displacement curve [18,19].

#### 3.1.6. Morphology of the Gels

The samples were prepared by freezing in liquid nitrogen for 10 min, and then freeze-dried for 24 h. The prepared samples were fractured in liquid nitrogen using a razor blade. The fractured samples were dried under a vacuum, attached to metal stubs, and sputter coated with gold under a vacuum for the SEM study. The interior and the surface morphology were observed under a scanning electron microscope (SEM, Hitachi S4800, Tokyo, Japan).

#### 3.1.7. Gel Stability

Stability of the gel formulations was also investigated. The organoleptic properties (color, odor), pH, drug content, and release profiles of the gels stored at 20 °C were examined on days 0, 15, 30 and 178. The performance of the hydrogels was not affected by the storage conditions, suggesting remarkable stability of the novel biomaterials under investigation.

#### 3.1.8. Studies of Equilibrium Swelling in the Hydrogels

A known weight of the dry gel was placed in a tea infusion bag and immersed in pH 4.0 and pH 9.0 buffer solutions, respectively, and kept at 25 °C for 48 h until equilibrium of swelling had been reached. The swollen gels were taken out and immediately weighed with a microbalance after the excess water on the surfaces was absorbed with a filter paper. The equilibrium swelling ratio (SR) was calculated using the following equation:

SR(%) = (*W*_s_ − *W*_d_)/*W*_d_ × 100

where *W*_s_ and *W*_d_ are the weights of the gels at the equilibrium swelling state and at the dry state, respectively [20]. Experiments were repeated in triplicate for each gel specimen and the mean value calculated. 

#### 3.1.9. Shear Bond Strength Tests for Dentin Bonding

Extracted non-carious, intact, human molars stored in water containing a few crystals of thymol at 4 °C were used within two months using protocol previously described by US [21,22,23]. 56 teeth samples prepared and divided into 7 groups of 8 each, A–F (Table 4) and stored in a solution of artificial saliva. These groups were then treated as outlined in Table 4. After 24 hours, a stud of each tooth was tested for shear bond strength. An Instron Universal Testing Machine at a crosshead speed of 0.5 mm/min was used to test the de-bonding strength. All data tests were analyzed using the non-parametric ANOVA test.

**Table 4:** Groups tested (8 teeth per groups).

Samples	Conditions
Group A	37% of phosphoric acid +primer+ Bonding immediately (negative control)
Group B	Self-etching primer + Bonding immediately (positive control)
Group C	Gel 1 + Bonding immediately
Group D	Gel 2 + Bonding immediately
Group E	Gel 3 + Bonding immediately
Group F	Gel 4 + Bonding immediately
Group J	Gel 5 + Bonding immediately

## 4. Conclusions

We evaluate the novel chitosan based functional drug delivery systems which can be successfully incorporated into “dual action bioactive restorative materials” capable to induce *in vitro* improved wound healing prototype and containing an antibiotic such as nystatin, krill oil as an antioxidant and hydroxyapatite as a molecular bone scaffold as function specific biomaterials capable of initiating bio-repair and bio-adhesion *in vitro* in a site specific manner.
